# Use of Probiotic Bacteria and Bacteriocins as an Alternative to Antibiotics in Aquaculture

**DOI:** 10.3390/microorganisms10091705

**Published:** 2022-08-24

**Authors:** Wellison Amorim Pereira, Carlos Miguel N. Mendonça, Alejandro Villasante Urquiza, Viggó Þór Marteinsson, Jean Guy LeBlanc, Paul D. Cotter, Elías Figueroa Villalobos, Jaime Romero, Ricardo P. S. Oliveira

**Affiliations:** 1 Microbial Biomolecules Laboratory, Faculty of Pharmaceutical Sciences, São Paulo University, Rua do Lago 250, Cidade Universitária, São Paulo 05508-000, SP, Brazil; 2 Facultad de Medicina Veterinaria y Agronomía, Universidad de Las Américas, Santiago 7500000, Chile; 3 Matís OHF, Microbiology Research Group, Vínlandsleið 12, 113 Reykjavík, Iceland; 4 Centro de Referencia para Lactobacilos (CERELA-CONICET), San Miguel de Tucuman T4000, Argentina; 5 Teagasc Food Research Centre, Moorepark, APC Microbiome Ireland, T12 K8AF Cork, Ireland; 6 Nucleus of Research in Food Production, Faculty of Natural Resources, Catholic University of Temuco, Temuco 4780000, Chile; 7 Laboratorio de Biotecnología de Alimentos, Instituto de Nutricion y Tecnologia de los Alimentos (INTA), Universidad de Chile, El Libano 5524, Santiago 783090, Chile

**Keywords:** probiotic, bacteriocin, antibiotic, aquaculture, biotechnology

## Abstract

In addition to their use in human medicine, antimicrobials are also used in food animals and aquaculture, and their use can be categorized as therapeutic against bacterial infections. The use of antimicrobials in aquaculture may involve a broad environmental application that affects a wide variety of bacteria, promoting the spread of bacterial resistance genes. Probiotics and bacteriocins, antimicrobial peptides produced by some types of lactic acid bacteria (LAB), have been successfully tested in aquatic animals as alternatives to control bacterial infections. Supplementation might have beneficial impacts on the intestinal microbiota, immune response, development, and/or weight gain, without the issues associated with antibiotic use. Thus, probiotics and bacteriocins represent feasible alternatives to antibiotics. Here, we provide an update with respect to the relevance of aquaculture in the animal protein production sector, as well as the present and future challenges generated by outbreaks and antimicrobial resistance, while highlighting the potential role of probiotics and bacteriocins to address these challenges. In addition, we conducted data analysis using a simple linear regression model to determine whether a linear relationship exists between probiotic dose added to feed and three variables of interest selected, including specific growth rate, feed conversion ratio, and lysozyme activity.

## 1. Introduction

There has been a growing global demand for animal protein, with fish representing a particularly important source. However, systematic and unbalanced human exploitation has led to an 80% reduction of the wild fish populations in the oceans. In parallel, the strong expansion of fish farming and aquaculture production has created a set of new challenges far beyond those involving the growth of the sector and its food supply chains [1]. To continue to grow, the aquaculture sector must focus on resolving difficulties through the demarcation of new breeding areas, accessing highly nutritious feed, developing new technologies and technical support, addressing logistic management limitations, and, very importantly, optimizing the ability to predict, avoid, and contain infections and diseases [2].

Fish consumption has grown in recent decades. It is estimated that a 3.2% increase occurred between 1961 and 2016, a figure that surpassed the corresponding rises in terrestrial animal protein production (2.8%). The estimated annual consumption per person has also increased significantly; for example, in 1961, average global consumption was less than 9 kilograms (kg), but by 2015, it had increased to 20.2 kg, with an additional growth from 20.3–20.5 kg estimated from 2016–2017 [3]. Most of production derived from aquaculture is intended for human consumption. By 2030, aquaculture is expected to be responsible for producing about 109 million tonnes for human consumption, compared with a predicted 74 million tonnes from exploratory fishing [3], a level of growth that is supported by low taxation levels [4,5]. However, many obstacles may hamper the predicted growth of aquaculture. Of these, the failure to predict and contain infections, diseases, and antibiotic resistance is the most perturbing [6].

As a strategy to minimize production losses due to infectious bacterial outbreaks, the use of antibiotics has been widely employed in recent decades [7]. However, their use is not sustainable and other options must be examined.

The objective of this review is to provide recent information relating to the importance of aquaculture in the animal protein production sector and its global economic impacts and growth prospects, as well as its present and future challenges generated by outbreaks and antimicrobial resistance, while highlighting the potential merits of employing probiotics and bacteriocins within this industry. Beneficial microorganisms (probiotics) and bacteriocins are novel solutions that could help reduce the use of antibiotics in aquaculture.

## 2. Antibiotics and Fish Infection Control

Along with their therapeutic applications to treat and control the spread of bacterial disease in juvenile and adult fish, antibiotics could be used as tools to avoid and prevent future infections beginning from the first days of fish development, when used as growth factors in feeding formulations [7]. This is sustained by farmers’ perception that the continuous presence of small doses of antibiotics in the fish growth environment helps to significantly reduce production costs. Due to the perception established between the decreased proliferation of pathogenic microorganisms with lower production losses and decreased time required to attain market weights, the abusive and unregulated use of these important therapeutic agents has expanded worldwide [7,8]

This is particularly worrying since, according to data reported by the World Health Organization (WHO) [9], a significant proportion of these antibiotics are also used as essential therapeutic agents for the treatment of bacterial diseases in humans. Therefore, the uncontrolled application of these antibiotics in animal protein production presents an enormous risk to human health [10]. Antibiotics can kill beneficial microorganisms, cause disturbances in the microbiota [11], affect nutrition and immunity [12], and their use can lead to the selection of resistant bacteria and the zoonotic transmission of resistance genes to the human microbiota [13]. Due to concerns relating to the global emergence of antibiotic resistance, global authorities and several developed countries, such as Canada, Japan, the United States, and members of the European Union, have implemented strict rules on the use of antibiotics in fish breeding [14]. Restrictions were officially approved, selecting a limited and smaller group of antibiotics that can be used in fish breeding, such as erythromycin, amoxicillin, florfenicol, oxytetracycline, oxolinic acid, flumequine, and combinations of sulphonamides [15]. Notably, a number of these antibiotics are considered essential for disease control in humans [9]. Even more importantly, these restrictions may have little impact globally as the majority of fish production is located in countries that have not adopted similar laws to regulate the use of antibiotics in animals. Thus, one can have extremes whereby, for example, Chile uses approximately 900 g of antibiotics for each tonne of fish while Norway uses only 0.17 g [14,16]. Furthermore, in Brazil, one of the top 25 aquaculture producers, many producers have increased the size of their production areas without following international standards of good environmental management practices. As a result, negative environmental effects and antibiotic-contaminated fish are common [17].

Ultimately, the continued extensive use of antibiotics by some countries is not sustainable, and as the number of bacterial disease outbreaks associated with the artificial environmental conditions of aquaculture increases and restrictive antibiotic use policies are implemented at an international level, new infectious control and prevention protocols are needed [7]. These new protocols are required to control the most common cause of fish diseases, i.e., bacterial infections. These include infections caused by *Aeromonas salmonicida* [15], *Vibrio anguillarum* [18], *Streptococcus agalactiae* [19], *Flexibacter columnaris* [20], *Aeromonas hydrophila* [21], *Aeromonas caviae* [22], *Pseudomonas aeruginosa* [23], *Enterococcus* spp. [24], *Francisella noatunensis* [25], and *Flavobacterium psychrophilum* [26].

Naturally, producers of non-antibiotic antimicrobials have received great attention as an alternative to the use of antibiotics [27]. In particular, probiotic microorganisms have been increasingly investigated as a means of improving fish defenses, especially as they are considered safe and are also frequently producers of antimicrobial peptides, such as bacteriocins [7].

## 3. Probiotic Use in Aquaculture

Probiotics are defined as live microorganisms that, when administered in adequate amounts, have the ability to confer health benefits on their host [28]. However, there is no consensus as to the value of applying probiotics to aquaculture. According to Wang et al. (2019), the way these animals relate to and are influenced by the environment is different from other animals, and so strains specifically tailored for aquaculture use need to be evaluated. Verschuere et al. (2000) proposed a new concept when defining probiotics for aquacultural use. Their concept differs from the standard definition of probiotics in that it suggests that probiotics for aquaculture use must have a beneficial action on both the host microbiota and the environment where the fish is located, optimizing the effect of food, animal health, and weight gain [29]. It is also important to note that chemical and physical factors, such as water quality (level of oxygen and carbon dioxide, temperature, pH, and presence of organic matter), fish density, or physical injury during handling, can lead to physiological reactions that culminate in the development of disease [30]. Furthermore, environmental changes or stress exposure can negatively affect fish development via immunosuppression. Thus, probiotic administration may also be targeted towards providing a protective response against these external stimuli [1].

Water and other living organisms might spread microorganisms from the gut microbiota of fish and probiotics. After reaching the host’s intestinal mucosa, these microorganisms perform vital functions. Several anatomical structures of aquatic animals are sites for the growth of microorganisms, such as the skin, gills, and especially the gastrointestinal tract [1,31]. Feces and intestinal mucus of fish are the main sources of microorganisms with probiotic potential. After isolation, these microorganisms are tested and can be used as a supplement in the feeding of aquatic animals [32]. The larval stage of growth is optimal with respect to probiotic use in aquaculture, and the consequences of early colonization of these microorganisms can be amplified throughout a fish’s life stages [33,34].

The probiotic microorganisms used in aquaculture have included specific strains of yeasts, algae, and especially bacteria, including representatives of *Bacillus* sp., *Lactococcus* sp., *Micrococcus* sp., *Carnobacterium* sp., *Enterococcus* sp., *Lactobacillus* sp., *Streptococcus*, and *Weissella* sp. [35]. Bacteria belonging to the group of LAB are considered GRAS, i.e., generally reported as safe [36] and can produce natural compounds with antimicrobial potential and also stimulate the immune system; thus, most probiotic studies are conducted with strains of LAB [37].

**Figure 1:**
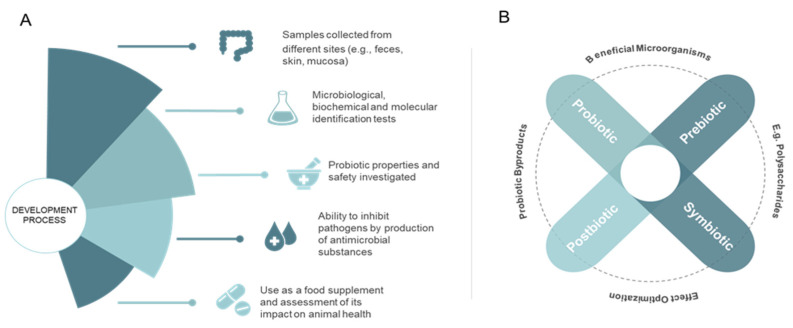
Probiotics development processes for feed and techniques to improve probiotic supplementation effects. (**A**) The different stages before probiotic bacteria use in aquaculture. From a sample, tests to identify genus and species are performed. Then, tests with and without the use of living organisms evaluate its properties and use as a food additive in animal feed. (**B**) In order to optimize aquaculture production processes, different techniques have been used. Probiotic microorganisms are those that confer benefits to the host; prebiotics are nondigestible food components that benefit the colonization of certain bacteria, such as probiotics; synbiotics are the combination of probiotics and prebiotics in the same product; mixtures of probiotics are prepared from the combination of more than one probiotic microorganism to potentiate their action; and postbiotics, dead probiotics or byproducts, are commonly associated with safety [38,39,40,42].

The use of probiotic microorganisms in experiments with aquatic animals has achieved promising results (Table 1), and feed supplementation effectiveness can be optimized if different approaches for the use of probiotics are tested (Figure 1) [38], including the use of mixtures of probiotics where complementary effects can be obtained. Supplementation with prebiotics, which are nondigestible food components that benefit colonization by providing nutrients and protection to probiotic and other desirable strains, or synbiotics, which are combinations of probiotics and prebiotics in the same product, can also have value [38,39,40]. Finally, postbiotics, which are the products of probiotic growth, including bacteriocins, can also have a key role [41].

## 4. Mode of Action and Benefits of Probiotic

**Figure 2:**
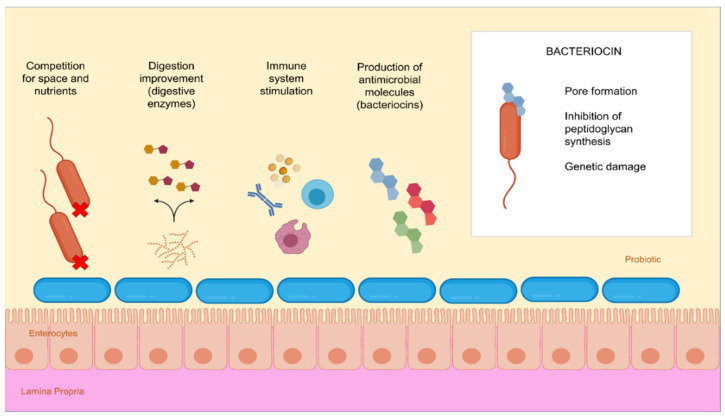
Probiotics and bacteriocins mode of action. Probiotics beneficial effects come from several mechanisms. They secrete digestive enzymes that contribute to macronutrients breakdown, increasing absorption by the host. They can act by blocking pathogens due to competition for space and nutrients, by stimulating the immune system (without the presence of disease) and via the production of antimicrobial substances (such as lactic acid and bacteriocins). Bacteriocins mode of action may vary according to their characteristics. They can lead to death via pore formation, preventing the action of peptidoglycan transporters and, consequently, cell wall synthesis, and via damage to genetic material and protein synthesis. Probiotics, bacteriocins, and the host nutritional improvement contribute to pathogens elimination and diseases control [121,122].

Among the studies that have demonstrated the benefits of probiotic use, different mechanisms of action have been noted, differing by species specificities and environmental conditions that the microorganism encounters [37,117]. Probiotics have been shown to be able to decrease lactose intolerance and infant diarrhea in humans, and many promising studies have shown that they can stimulate the immune system and prevent numerous diseases, including mucosal inflammation, obesity, diabetes, heart and neurological diseases, and certain types of cancer. In this current review, the focus will be placed on the prevention of pathogenic microorganisms in aquacultural settings. Beneficial strains can function by blocking pathogenic microorganisms due to competition for space on host cell surfaces (Figure 2) [118]. Probiotic use in feed improves the health of aquatic animals and no negative effects have been observed after consumption [14]. Strains of *Lactobacillus* are commonly recommended for aquaculture, and dietary supplementation results in better enzyme activity, immune response, development, weight gain, and even water quality improvement [32,119]. The stimulation of digestive enzyme production, such as amylase, protease, lipase, and lysozyme, can be an important consequence of probiotic use [118]. In healthy animals, these enzymes are intrinsically associated with improved digestibility, nutritional intake, and weight gain [120]. Improving the digestibility of certain compounds may reduce blood lipid rates and even address problems arising from the intolerance to certain compounds [32].

The benefits of probiotics in aquaculture extend beyond animal health and can also be used to improve water quality. The accelerated fish production process creates a stressful environment favorable to pathogenic microorganisms and diseases. However, probiotic use in fish farm systems can modify the aquatic environment and, by reducing the populations of undesirable microorganisms, reduce the chances of disease development [123].

In this review, we conducted data analysis using a simple linear regression model (GraphPad Prism version 9.0, GraphPad Software, San Diego, CA, USA) to determine whether a linear relationship between probiotic dose added to feed and three variables of interest selected, including specific growth rate (SGR; 38 studies), feed conversion ratio (FCR; 32 studies), and lysozyme activity (8 studies), exists. For analysis purposes, we have only taken into account the presence or absence of probiotics without considering the type of probiotic as well as whether they were used as single or multiprobiotic treatment.

**Figure 3:**
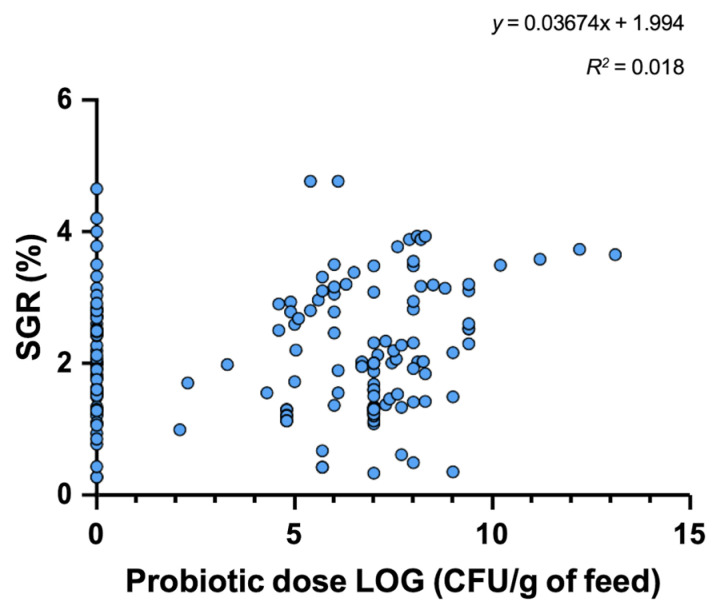
Data analysis revealed no significant correlation between probiotic dose in feed and SGR in fish. The circles represent the mean of experimental groups (n = 3; either control group or probiotics treatment group) tested in the studies considered for the regression analysis.

**Figure 4:**
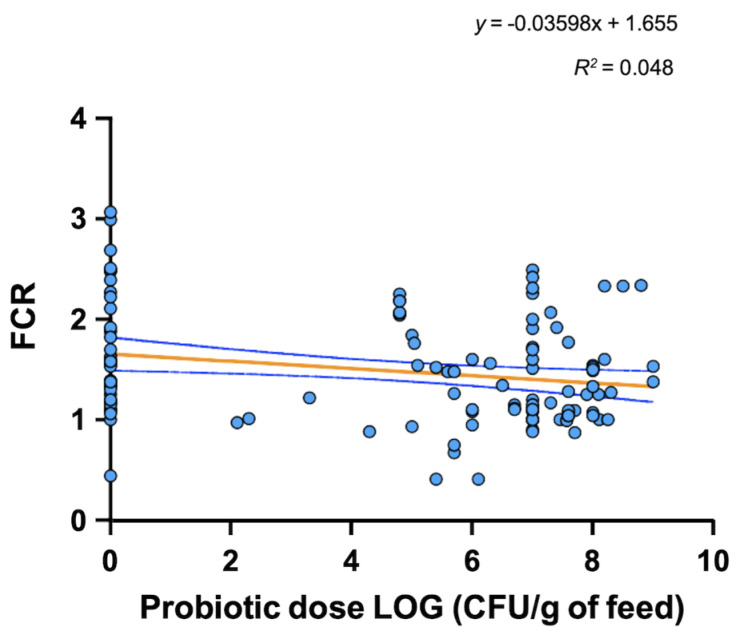
Data analysis revealed significant correlation between probiotic dose in feed and FCR. The circles represent the mean of experimental groups (n = 3; either control group or probiotics treatment group) tested in the studies considered for the regression analysis.

**Figure 5:**
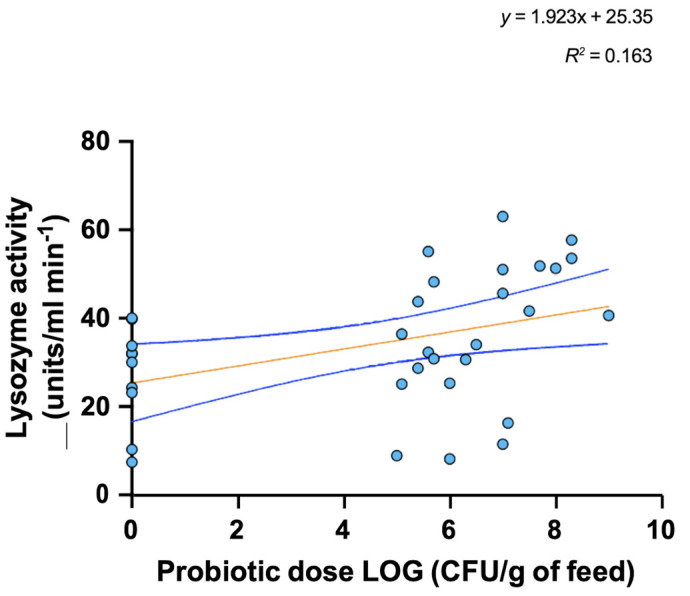
Data analysis revealed significant correlation between probiotic dose in feed and Lysozyme activity. The circles represent the mean of experimental groups (n = 3; either control group or probiotics treatment group) tested in the studies considered for the regression analysis.

Probiotic dose added to feed was transformed to log10 for graphic representation purposes. Data analysis revealed no significant correlation (*p* = 0.085) between probiotic dose in feed and SGR in fish (*R*^2^ = 0.0182; Figure 3). However, we detected a significant correlation (*p* = 0.014; *p* = 0.017) between probiotic dose in feed and FCR as well as lysozyme activity (*R*^2^ = 0.048; *R*^2^ = 0.163, respectively; Figure 4 and Figure 5) in fish. These results suggest adding probiotics to the diet improves the utilization efficiency of feed in fish and thus contributes to improving the economy and well-being of fish farming. This is especially true since feed is considered to be the highest cost in aquaculture facilities, particularly in intensive culture systems where feed costs represent close to 50% of the variable production cost [124].

The improvement in fish feed utilization could be a consequence of probiotic microbes contributing directly or indirectly, via induced changes in gut microbiota composition, to metabolize undigested nutrients via microbial enzyme activity. However, an enhancement of nutrient absorption surface/capacity due to a stimulatory effect of probiotic microbes on gut epithelium development and gut health might contribute to this outcome as well. For example, short chain fatty acids (SCF) derived from probiotic metabolism influence epithelial cell metabolism, helping with busting diverse energy-demanding cellular processes in enterocytes, such as producing mucin and tight junction enterocyte proteins, which contribute to the integrity of the intestinal barrier [125].

For its part, our analysis revealed that SGR was not affected by adding probiotics to the diet of fish. A possible explanation of this lack of significance is due to the exponential function of SGR, showing some imprecision when determining fish growth efficiency using either long-term data or data over different life stages. Thus, SGR should be used when fish are exactly of the same age, since the growth performance of fish during different life stages introduces a bias into the calculation. Because the studies included in our analysis covered different life stages and trial periods, SGR may have been an unsuitable mathematical model for comparing growth performed in these heterogenous data analysis environments [126].

Finally, the significant positive correlation between lysozyme activity and probiotic dose added to feed found across the studies included in the analysis supports the idea that probiotics provide health benefits to fish (Figure 5). Lysozyme is a hydrolytic glycosidase [(β-) glycoside hydrolase that exerts several important functions related to innate immunity, including the lyse of Gram-positive and Gram-negative bacterial cell membranes (acting as an antimicrobial agent) and activation of the complement system and phagocytes. It is ubiquitously distributed in several tissues, mucus, lymphoid tissue, plasma, and other body fluids [127]. Hence, increasing lysozyme activity by adding probiotics to feed might play an important role in enhancing fish disease resistance in intensive culture systems.

## 5. Bacteriocin Use in Aquaculture

In recent years, bacteriocins have received substantial attention as antimicrobial compounds. Although bacteriocins have been predominantly used as food preservatives, they are now receiving better attention as potential clinical antimicrobials and as possible immune-modulating agents. Hence, bacteriocin use is another important strategy to control antibiotic-resistant bacteria and improve health [121]. Bacteriocins are a heterogeneous group of small, ribosomally-synthesized antimicrobial peptides. They can have a wide variety of producers, spectrums of action (Figure 2), and biochemical properties [121,128].

Since 1925, with the discovery of colicin, research on bacteriocins has received considerable attention [129], and by 1995, more than a hundred different types of bacteriocins had been identified [130]. Bacteriocins can provide an important competitive advantage for the species that produce them [131]. Probiotics of interest can produce bacteriocins at their site of action [132].

Several classes of bacteriocins have been evaluated [133]. Many of the bacteriocins tested for food-related applications are isolated from LAB [131]. These include nisin, which is produced by *L. lactis* and has been widely used as a food preservative for more than fifty years [134,135]. Others, such as pediocin PA-1, produced by *Pediococcus acidilactici* have been extensively studied due to their activity against Listeria monocytogenes in meat and dairy products [131]. Bacteriocins have also been investigated for their pharmaceutical application [129] because they could serve as a possible alternative to antibiotics to combat pathogenic microorganisms in live organisms [121]. As production losses in aquaculture due to bacterial diseases and bacterial resistance to antibiotics have increased [7,121], bacteriocins have been applied in aquaculture production systems due to their antimicrobial proprieties (including Gram-positive/Gram-negative inhibition) (Table 2). However, the application of probiotics and bacteriocins in fish feed supplementation requires rigorous testing to avoid any unexpected effects. Safety is essential to current research progress [136].

## 6. Safety

It is important that probiotics be properly developed and that new products be verified using validated scientific research. In some countries, probiotics have been approved for use based only on initial tests that generally attest to their antimicrobial and immunostimulatory activity. Furthermore, in 2017, during inspections by the US FDA (Food and Drug Administration, Silver Spring, MD, USA), more than 50% of the establishments visited in the probiotic industry had serious violations, all related to failures during the development process, including misidentification and even contamination of supplements, which compromises product efficacy and safety [136].

The transfer of resistance genes to the host microbiota is another growing concern that could result in a loss of commercial interest. In an in vitro experiment, it was observed that Lactobacillus plantarum M345 was able to transfer a resistance gene to Listeria monocytogenes [152]. In 2005, it was reported that a probiotic product that was approved by the FDA contained a strain with resistance to an important clinical antibiotic (tetracycline) and that the gene could be transmitted [136]. The presence of resistance genes in probiotics has already been described in the literature and has been studied. As one of the main advantages of using probiotics is their safety, it is necessary to pay more attention to this problem. If not controlled, it can represent a loss of consumer interest and economic losses to the sector [120].

However, it is important to emphasize that health problems resulting from the use of probiotics are very rare, both for animals and for humans. These microorganisms are already part of the host’s microbiota and any problems related to the use of probiotics are generally related to host immunity and other pre-existing diseases [153]. In addition, many countries already have very strict laws that ensure that the development and sale of probiotic products takes place safely [4,154].

## 7. Conclusions and Future Perspectives

Bacterial disease outbreaks in aquaculture systems have increased in the last few decades, and policies that restrict antibiotic use have been implemented. To avoid production losses, new therapeutic fish farming technologies and new infectious control and prevention protocols are required. The benefits of specific probiotics and bacteriocins which trigger directly or enhance the immune structure of aquatic species with respect to fish health and controlling pathogenic bacteria in aquaculture are clear. Further advancements in this area have the potential to cause a paradigm shift in aquaculture, resulting in higher quality foods, improved consumer health, increased sustainability (including environmental sustainability), and increased economic value.

## Figures and Tables

**Table 1:** Overview of probiotic effects on fish health or against aquaculture pathogenic bacteria.

Aquatic Specie	Probiotic	Pathogen or Challenge	Clinical Impact	Reference
*Oreochromis niloticus*	Mixture of LAB	*Trichodina* sp.	Improved growth rate and antiparasitic activity	[43]
*Cyprinus carpio*	*Pediococcus pentosaceus*	*Aeromonas hydrophila*	Probiotic increases digestive enzyme activity; enhancement of growth rate and immune response; resistance against bacterial infection	[44]
*Litopenaeus vannamei*	Mix of commercial probiotics (e.g., *Bacillus* spp., *Lactobacillus* spp., *Saccharomyces* spp.)	Not evaluated	The probiotics did not change water quality or growth parameters when compared with control group	[45]
*Salmonids*	*Vibrio alginolyticus*	*A. salmonicida,* *V. anguillariim,* *V. ordalii*	Pathogen inhibition	[46]
*Salmo salar*	*Tetraselmis suecica*	*A. salmonicida,* *S. liquefaciens,* *V. anguillariim,* *V. salmonicida*, *Y. ruckeri*	Suppress pathogen growth	[47]
*Salmo tutta*	*Lactococcus lactis, Leuconostoc mesenteroides*	*Aeromonas salmonicida*	Higher survival rate	[48]
*Mystus cavasius*	*Saccharomyces cerevisiae*	*Pseudomonas fluorescens*	Better weight gain, low mortality; resistance against tested pathogen	[49,50]
*Labeo rohita*	Probiotic mixture (*Bacillus subtilis, Pediococcus acidilactici,* yeast *Saccharomyces cerevisiae*) and symbiotics (*Bifidobacterium, Lactobacilli, Saccharomyces cerevisiae,* microalgae *Spirulina* sp., phytase)	Not evaluated	Better survival and growth rate; probiotic action is best if administered to developing fish in their first days	[50]
*Litopenaeus vannamei*	*Bacillus subtilis*	Not evaluated	Significant secretion of hepatopancreatic metabolites; expression of genes linked to antioxidant enzymes	[51]
*Oreochromis niloticus*	*Aspergillus oryzae*	*Aeromonas hydrophila*	Improvement of immune response and growth rate	[52]
*Oreochromis niloticus*	*Lactobacillus plantarum* L-137	Exposition to deltamethrin toxicity	Reduction of the toxicity	[52]
*Pagrus major*	*Pediococcus pentosaceus*	Not evaluated	Increased weight gain, mucus secretion, growth rate, bacterial resistance, and blood parameters	[53]
*Pagrus major*	*Lactobacillus plantarum*	Not evaluated	Immunostimulant property (innate defenses)	[54]
*Pagrus major*	*Lactobacillus rhamnosus* and *Lactococcus lactis*	Not evaluated	Better growth, feed utilization, serum lysozyme activity, bactericidal property, and lower triglycerides and cholesterol	[55]
*Oreochromis niloticus*	*Bacillus subtilis* and *Bacillus licheniformis*	Not evaluated	Enhanced immunological parameters (hematocrit, total leukocytes count, monocytes, and globulin), improved growth and feed utilization	[56]
*Oreochromis niloticus*	*Lactobacillus* sp., *Bacillus* sp., *Bifidobacterium* sp. (probiotic mixture)	Not evaluated	Antimicrobial activity, better growth rate	[57]
*Oreochromis niloticus*	*Lactobacillus plantarum*	*Enterococcus faecalis*	Modulation of gut microbiota, immune response, and resistance against pathogenic bacteria	[58]
*Atlantic salmon*	*Candida utilis*	*Chlorella vulgaris*	Counteracts intestinal inflammation	[59]
*Salmon salar*	Lactic acid bacteria	*Aeromonas salmonicida*	Higher mortality	[60]
*Gadus morhua* (Atlantic cod),	*Carnobacterium divergens*	*V. anguillarum*	Disease resistance	[61]
*Cyprinus carpio*	*Pseudomonas aeruginosa*	*Aeromonas hydrophila*	Antioxidant and immune action; better infection control with probiotic treatment	[62]
*Oreochromis mossambicus*	*Bacillus licheniformis* Dahb1 (105 and 107)	*Aeromonas hydrophilain*	Weight and specific growth rate improvement; high mucosal activity of enzymes; resistance to the infection	[63]
*Pangasius hypophthalmus*	*Bacillus licheniformis*	*Vibrio parahaemolyticus*	Increased immune, antioxidant and growth parameters; protected against infection	[64]
*Ctenopharynodon idellus*	*Bacillus subtilis*	*Aeromonas hydrophila, Aeromonas punctata, Edwardsiella ictaluri, Aeromonas punctate, Vibrio flurialis* and *Streptococcus agalactiae*	Inhibitory activity against all pathogenic bacteria tested	[65]
*Cyprinus carpio*	*Paenibacillus polymyxa*	*Aeromonas hydrophila*	Improved survival rate and immune response; disease resistance against pathogenic bacteria tested	[66]
*Litopenaeus vannamei*	*Bacillus subtilis, Bacillus pumilus, Bacillus tequilensis, Enterococcus faecalis*	Not evaluated	Significant difference in growth rate, weight gain, and survival	[67]
*Acipenser baerii*	*Lactobacillus* spp. *Bacillus subtilis, Bifidobacterium bifidum* (probiotics mixture)	Not evaluated	Immunity and growth improvement	[68]
*Oreochromis niloticus*	*Bacillus licheniformis*	*Streptococcus iniae*	Better survival rate	[69]
*Heteropnuestes fossilis*	*Bacillus subtilis*	*Aeromonas hydrophila* and *Aphanomyces invadans*	Bacterial treatment leads to a health improvement; fungi treatment does not	[70]
*Oncorhynchus mykiss*	*Lactobacillus rhamnosus*	*Yersinia ruckeri*	Improved growth rate, immune response, and antioxidant activity; pathogen inhibition	[71]
*Litopenaeus vannamei*	*Lactobacillus plantarum* and galactooligosaccharide (symbiotic)	*Vibrio harveyi* and *Photobacterium damselae*	Improvement in growth and health parameters; infection control; significant changes in intestinal microbiota of shrimp	[72]
*Salmonids*	*Carnobacterium Inhibens* K1	*Vibrio anguillarum*, *Aeromonas salmonicida*	Suppress pathogen growth	[73]
*Oreochromis niloticus* and *Cyprinus carpio*	*Lactococcus lactis* subsp. *lactis*, *Lactobacillus plantarum*, *Lactobacillus brevi*	*Vibrio* sp., *Staphylococcus* sp., *Pseudomonas aeruginosa, Salmonella enterica, Listeria monocytogenes*	Antimicrobial action	[74]
*Cyclopterus lumpus*	*Aliivibrio* sp.	*Moritella viscosa* (contamination)	Resistance against infection caused by *M. viscosa*; low incidence of mortality and ulcers	[75]
*Oreochromis niloticus*	*Bacillus velezensis, Bacillus subtilis, Bacillus amyloliquefaciens*	*Aeromonas hydrophila*	Improvement of immune response; antimicrobial activity	[76]
*Paralichthys olivaceus*	*Bacillus* sp. and β-glucan (symbiotic)	*Edwardsiella tarda*	Strain has significant antimicrobial activity; symbiotic effect improved growth performance; resistance against tested pathogen (antibiotic replacement)	[77]
*Apostichopus japonicus*	*Metschnikowia* sp.	Not evaluated	High activity of lysozyme, total nitric oxide synthase, trypsin, and phenoloxidase	[78]
*Lates calcarifer*	*Lactobacillus casei, Lactobacillus plantarum, Lactobacillus pentosus, Lactobacillus fermentum, Enterococcus faecium, Bacillus subtilis*, and *Saccharomyces cerevisiae*	*Aeromonas hydrophila*	The probiotic mixture improved growth and health status of Asian Seabass	[79]
*Oplegnathus fasciatus*	*Bacillus subtilis* E20	*Vibrio alginolyticus*	Better growth rate and immune response; pathogen resistance	[80]
*Salmon salar*	*Pediococcus acidilactici*	IPN virus	Antiviral response	[81]
*Pangasius bocourti*	*Bacillus aerius* B81	*Aeromonas hydrophila, Streptococcus agalactiae*	Antimicrobial effect against tested pathogens, high immune response	[82]
*Oreochromis niloticus*	*Lactobacillus plantarum*	Environmental challenges	High mucosal immune response	[83]
*Oncorhynchus mykiss*	*Lactobacillus acidophilus*	*Lactococcus garvieae*	Better growth rate, digestive enzyme production, resistance against tested pathogen	[84]
*Cyprinus carpio*	*Lactobacillus casei,* β-glucan and mannan oligosaccharide (symbiotic)	*Aeromonas hydrophila*	Symbiotic improves the digestibility; elevation in important enzymes (lipase, amylase, trypsin, and protease); low mortality	[85]
*Haliotis midae*	*Vibrio midae*	Not evaluated	Increase in growth performance and survival rate	[86]
*Labeo rohita*	*Bacillus* sp.	*Aeromonas hydrophila*	Improved hematological serum an immunological parameter	[87]
*Oncorhynchus mykiss*	*Gordonia bronchialis*	Not evaluated	Enhanced growth performance	[88]
*Penaeus indicus*	*Bacillus subtilis*	*Bacillus* sp., *Pseudomonas* sp., *Vibrio* sp., *Micrococcus* sp.	High bacteriocin production; diet with bacteriocin enhances shrimp growth; antibiotic potentials (well diffusion method)	[89]
*Salmon salar*	*Carnobacterium divergens*	*Aeromonas salmonicida,* *Vibrio anguillarum*	Prevent pathogen-induced damage	[90]
*Salmon salar*	*Methylococcus capsulatus*	Not evaluated	No inflammation with soybean meal	[91]
*Oncorhynchus mykiss*	*Enterococcus casseliflavus*	*Streptococcus iniae*	Elevated digestive enzyme activity, humoral immunity (IgM), total serum protein, and albumin production	[92]
*Salmon salar*	*Lactobacillus delbruckii*	*Aeromonas salmonicida*	Prevent pathogen damage	[93]
*Oreochromis niloticus*	*Bacillus* sp.	*Aeromonas hydrophila, Micrococcus luteus, Pseudomonas fuorescence, Enterococcus faecalis*, and *Streptococcus agalactiae*	Probiotic potential (resistance to adverse stomach condition, production of important enzymes)	[94]
*Etroplus suratensis* and *Oreochromis Mossambicus*	*Bacillus* sp., *Micrococcus* sp.	Not evaluated	Better growth performance and nutritional efficiency	[95]
*Danio rerio*	*Bacillus subtilis* (transgenic probiotic)	Not evaluated	The transgenic probiotic (phytase) can improve fish nutrition	[96]
*Dicentrarchus labrax*	*Vibrio lentus*	Not evaluated	Immunomodulation and activation of genes associated to cell proliferation	[97]
*Oreochromis niloticus*	*Bacillus amyloliquefaciens*	*Yersinia ruckeri, Clostridium perfringens*	Improved immune status (IL-1 and TNF-α mRNA) and disease resistance	[98]
*Litopenaeus vannamei*	*Enterococcus faecium* and *Lactobacillus pentosus*	*Vibrio harveyi, Vibrio parahaemolyticus*	High antibacterial activity and survival rate; improved humoral immune response	[99]
*Oncorhynchus mykiss*	*Lactobacillus plantarum*	*Yersinia ruckeri*	High activity of lysozyme and alkaline phosphatase; no interference in the production of immunological proteins	[100]
*Oreochromis niloticus*	*Enterococcus faecium*	*Aeromonas hydrophila*	Better growth rate and immune defenses	[101]
*Oreochromis niloticus*	*Bacillus* sp.	Streptococcosis (*Streptococcus agalactiae*)	Controlled the Streptococcosis caused by pathogenic bacteria tested	[102]
*Rutilus caspicus*	*Enterococcus faecium*	*Aeromonas hydrophila, Yersinia ruckeri*	Better growth rate, immune response, and pathogen resistance	[103]
*Ictalurus punctatus*	*Bacillus velezensis*	Not evaluated	Induction of growth in fingerling and water quality improvement	[104]
*Litopenaeus vannamei*	*Bacillus subtilis*	Not evaluated	Better growth performance and feed utilization	[105]
*Carassius auratus*	*Enterococcus faecium*	*Aeromonas hydrophila*	High survival rate as a result of *E. faecium* probiotic proprieties; quorum sense potential	[106]
*Atlantic salmon*	*Pediococcus acidilactici*		Improvements in the gut health	[107]
*Oncorhynchus mykiss*	*Lactobacillus fermentum, Lactobacillus buchneri, Saccharomyces cerevisiae* (probiotics mixture)	Not evaluated	Immunity improvement	[108]
*Danio rerio*	*Pseudomonas aeruginosa*	*Vibrio parahaemolyticus*	Reduced mortality, inhibited biofilm, high level of phagocytic cells, superoxide dismutase activity, and lysozyme	[109]
*Oreochromis niloticus*	*Bacillus cereus, Alcaligenes faecalis*	Environmental challenges	High production of immune proteins and decrease of phosphorus water concentration	[110]
*Ctenopharyngodon idellus*	*Shewanella xiamenensis* and *Aeromonas veronii*	*Aeromonas hydrophila*	Enhancement of phagocytic, lysozyme activity, and expression of immune genes	[111]
*Rhamdia quelen*	*Lactococcus lactis*	*Aeromonas hydrophila, Streptococcus agalactiae*	Antimicrobial activity against tested pathogens	[112]
*Carassius auratus*	*Bacillus velezensis*	*Aeromonas hydrophila*	Improved survival rate and immune response	[113]
*Nile tilapia*	Probiotic mixture	Aluminum exposition	Probiotics regulated gut microbiota structure and function	[114]
*Oreochromis niloticus*	*Lactobacillus plantarum*	Aluminum intoxication	Enhanced feed utilization and growth; decreased deaths caused by aluminum and its accumulation	[115]
*Ctenopharyngodon idellus*	*Bacillus paralicheniformis*	Not evaluated	High adhesion and colonization capacity	[116]

**Table 2:** Overview of bacteriocin effects in fish health or against aquaculture pathogenic bacteria.

Aquatic Specie	Bacteriocin	Pathogen or Challenge	Clinical Impact	Reference
*Epinephelus areolatus*	CAMT2	*Listeria monocytogenes, Staphylococcus aureus*	Antimicrobial activity against tested pathogens	[137]
*Labeo rohita*	Bacteriocin produced by *Bacillus subtilis LR1*	*Aeromonas hydrophila, Aeromonas salmonicida, Bacillus mycoides, Pseudomonas fluorescens*	In vitro antimicrobial activity against tested pathogens	[138]
*Oncorhynchus tshawytscha*	Enterocina AS-48	*Lactococcus garvieae*	Antimicrobial activity against tested pathogen (in vitro and in vivo)	[139]
*Penaeus monodon*	Bacteriocin 99% homologous to that produced by *Bacillus* sp.	*Vibrio alginolyticus, Aeromonas hydrophila, Pseudomonas stutzeri*	In vitro inhibitory activity against tested pathogens	[140]
*Pseudosciaena croce*	Coagulina L1208	*Escherichia coli, Shewanella putrefaciens, Staphylococcus aureus*	Bacteriostatic antimicrobial activity against tested pathogens	[141]
*Litopenaeus vannamei*	Bacteriocin produced by *Lactobacillus plantarum* FGC-12	*Vibrio parahaemolyticus*	Pathogen inhibition	[142]
*Perca* sp., *Tuna* sp., *Platax* sp.	PSY2	*Listeria monocytogenes*	In vitro pathogen inhibition; possible biopreservative against degradation	[143]
*Odontesthes platensis*	Mundticin KS	*Pseudomonas aeruginosa,* *S. putrefaciens*	In vitro antimicrobial activity against tested pathogen and Gram-positive bacteria	[144]
*Odontesthes platensis*	Nisin Z	*Lactococcus garvieae*	Pathogen growth inhibition	[145]
Fermented fish roe	Bacteriocin produced by *Enterococcus faecium* CN-25	*Listeria monocytogenes*	In vitro pathogen inhibition	[146]
*Tilapia* sp., *Catla catla, Cyprinus carpio*	Bacteriocin isolated from *Pediococcus acidilactici*	*Listeria monocytogenes*	In vitro antimicrobial activity against tested pathogen	[147]
*Acipenseridae, Oncorhynchus clarkii*	Plantaricin LPL-1	*Listeria monocytogenes*	In vitro antimicrobial activity against tested pathogen and Gram-positive bacteria	[148]
*Pangasius bocourti*	7293	*Listeria monocytogenes, Staphylococcus aureus, Aeromonas hydrophila, Escherichia coli, Pseudomonas aeruginosa, Salmonella* Typhimurium	Gram-positive and Gram-negative growth inhibition	[149]
*Oxyeleotris lineolata*	L49	*Streptococcus iniae*	In vitro antimicrobial activity against tested pathogen	[150]
*Mimachlamys nobilis*	PE-ZYB1	*Listeria monocytogenes*	In vitro antimicrobial activity against Gram-positive and Gram-negative bacteria; pathogen inhibition	[151]
*Litopenaeus vannamei*	Nisin	*Listeria monocytogenes*	Antimicrobial activity against tested pathogen (in vitro and in vivo)	[135]

## Data Availability

The data that support the results of this study are available from the corresponding author upon request.
